# Evolutionary dynamics of tree invasions: complementing the unified framework for biological invasions

**DOI:** 10.1093/aobpla/plw085

**Published:** 2016-12-30

**Authors:** Rafael D. Zenni, Ian A. Dickie, Michael J. Wingfield, Heidi Hirsch, Casparus J. Crous, Laura A. Meyerson, Treena I. Burgess, Thalita G. Zimmermann, Metha M. Klock, Evan Siemann, Alexandra Erfmeier, Roxana Aragon, Lia Montti, Johannes J. Le Roux

**Affiliations:** 1 Setor de Ecologia, Departamento de Biologia, Universidade Federal de Lavras, Lavras, MG, Brazil. Programa de Pós-Graduação em Ecologia Aplicada, Caixa Postal 3037, Lavras, MG, CEP 37200-000, Brazil; 2 Bio-protection Research Centre, Lincoln University, Lincoln, 7647, New Zealand; 3 Forestry & Agricultural Biotechnology Institute, University of Pretoria, Pretoria, South Africa; 4 Centre for Invasion Biology, Department of Botany & Zoology, Stellenbosch University, Private Bag X1, Matieland, 7602, South Africa; 5 Centre for Ecology, Evolution and Environmental Changes, Faculty of Sciences, University of Lisbon, Campo Grande, Lisbon 1749-016, Portugal; 6 Department of Natural Resources Science, University of Rhode Island, Kingston, RI, USA; 7 School of Veterinary and Life Sciences, Murdoch University, Murdoch 6150, Australia; 8 Instituto de Pesquisas Jardim Botânico do Rio de Janeiro, Laboratório de Sementes, Rio de Janeiro, RJ, Brazil; 9 Department of Biological Sciences, Louisiana State University, Baton Rouge, Louisiana, USA; 10 Biosciences Department, Rice University, Houston, TX, USA; 11 Institute for Ecosystem Research, Kiel University, Germany; 12 Instituto de Ecologia Regional, Facultad de Ciencias Naturales, Universidad Nacional de Tucumán, CONICET, Tucuman, Argentina

**Keywords:** Contemporary evolution, epigenetics, evolution, genetic variation, invasion biology, second-genome, tree invasions

## Abstract

Evolutionary processes greatly impact the outcomes of biological invasions. An extensive body of research suggests that invasive populations often undergo phenotypic and ecological divergence from their native sources. Evolution also operates at different and distinct stages during the invasion process. Thus, it is important to incorporate evolutionary change into frameworks of biological invasions because it allows us to conceptualize how these processes may facilitate or hinder invasion success. Here, we review such processes, with an emphasis on tree invasions, and place them in the context of the unified framework for biological invasions. The processes and mechanisms described are pre-introduction evolutionary history, sampling effect, founder effect, genotype-by-environment interactions, admixture, hybridization, polyploidization, rapid evolution, epigenetics and second-genomes. For the last, we propose that co-evolved symbionts, both beneficial and harmful, which are closely physiologically associated with invasive species, contain critical genetic traits that affect the evolutionary dynamics of biological invasions. By understanding the mechanisms underlying invasion success, researchers will be better equipped to predict, understand and manage biological invasions.

## Teaser

Evolution greatly impacts the outcomes of biological invasions. In our review, we review such evolutionary processes, with an emphasis on tree invasions, and place them in the context of a unified framework for biological invasions. The processes and mechanisms described are pre-introduction evolutionary history, sampling effect, founder effect, genotype-by-environment interactions, admixture, hybridization, polyploidization, rapid evolution, epigenetics, and second-genomes. By understanding the mechanisms underlying invasion success, researchers will be better equipped to predict, understand, and manage biological invasions.

## Introduction 

Evolutionary processes are important mechanisms contributing to successful biological invasions. An extensive body of research demonstrates that invasive populations often undergo phenotypic and ecological divergence from their native sources (Williams 2009; Williams *et al.* 2010). Some lineages or genotypes are highly successful invaders, whereas others may fail to invade (Lachmuth *et al.* 2010; Meyerson *et al.* 2010; Corliss and Sultan 2016). After introduction, invasive populations originating from the same sources may colonize different environments in the introduced range (Zenni *et al.* 2014; Zenni and Hoban 2015) and, in some instances, adaptation can be surprisingly rapid (Colautti and Barrett 2013). During the invasion process, evolutionary factors can operate at different invasion stages, e.g. introduction, establishment and spread (Blackburn *et al.* 2011). It is therefore important to incorporate these factors into frameworks of biological invasions in order to understand the roles that ecological versus evolutionary drivers play in invasion success.

Invasive trees have significant environmental and economic impacts worldwide (Richardson and Rejmánek 2011; Rejmánek *et al.* 2013; Valduga *et al.* 2016), but our understanding of the drivers and the extent of genetic changes in invasive tree populations are limited relative to other plant functional groups such as herbaceous plants and shrubs. To date, studies have more commonly focused on the ecological and anthropogenic, rather than on evolutionary drivers of tree invasions (Lamarque *et al.* 2011; Procheş *et al.* 2012). Given that the life-history traits of trees include long lifespans and generation times, it is not surprising that their evolutionary dynamics are still poorly understood. However, many tree invasions pose unique natural experiments—e.g. introductions of diverse provenances for forestry and selective breeding for yield—to test the importance of evolutionary factors in promoting invasions.

Here, we review the evolutionary mechanisms associated with tree invasions and place them in the context of the unified framework for biological invasions (Blackburn *et al.* 2011). An increasing number of studies suggest several evolutionary mechanisms allow populations to overcome different barriers of the invasion process: the introduction–naturalization–invasion continuum (Richardson  and Pyšek 2012). Consequently, it is imperative that such evolutionary aspects of the invasion process are placed within a unified framework. In considering evolutionary mechanisms associated with tree invasions, we take a broad view and include not only the plant genome but also the epigenome and the genomes of closely associated plant symbionts (including mutualists and pathogens), recently been referred to as the ‘second-genome’ (Zhao 2010; Zhu *et al.* 2010; Grice and Segre 2012). This broader view allows us to include all of the genetic and potentially heritable traits of an organism (although the second-genome may be partially acquired from the new environment). In this regard, it has been suggested that altered second-genome interactions are one of the most important aspects defining how introduced species differ from native species (Dickie *et al.* 2016). 

## The unified framework

**Figure 1:**
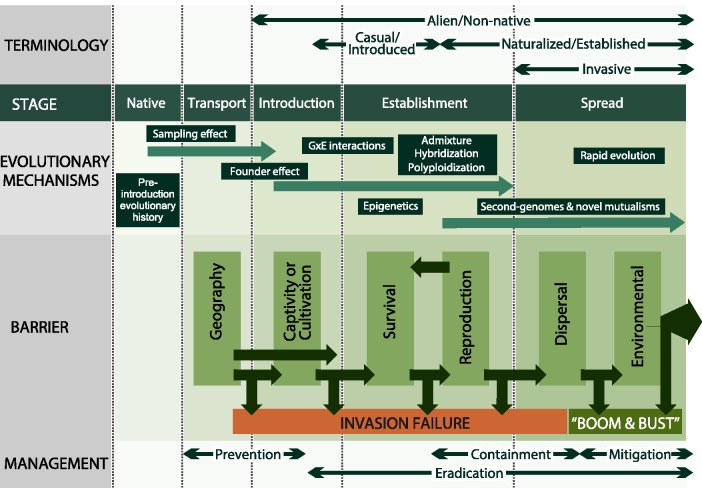
The unified framework for biological invasions (Blackburn *et al.* 2011) expanded to incorporate evolutionary mechanisms associated with invasions, including evolutionary effects occurring in the species’ native ranges.

The unified framework proposed by Blackburn *et al.* (2011) integrates two widely adopted frameworks aimed at conceptualizing the invasion process (Williamson 1996; Williamson and Fitter 1996; Richardson *et al.* 2000). The unified framework mainly focuses at the population-level and combines a suite of barriers (geography, captivity/cultivation, survival, reproduction, dispersal and environmental) and their underlying stages (transport, introduction, establishment and spread) that a species must overcome in order to become invasive (Fig. 1). Importantly, it also includes those cases where invasions fail or where populations undergo ‘boom and bust cycles’. The framework, however, does not describe specific mechanisms allowing transitions from stage to stage, mechanisms hindering transitions or mechanisms causing failures (*sensu* Zenni and Nuñez 2013).

Box 1. Research example: forestry provenance trials as tools for understanding evolutionary dynamics of tree invasions

Invasions are ‘natural experiments’ where researchers can study multiple ecological and evolutionary factors over long periods of time and large spatial scales (Sakai *et al.* 2001; Yoshida *et al.* 2007). However, while invasions can provide long-term large-scale data sets that in many instances are virtually impossible to obtain from manipulative experimental systems (Bøhn *et al.* 2008), often they also have numerous sources of biases and lack critical information about historical factors, initial sources, dispersal pathways, and state of the habitat at the time of arrival (Richardson *et al.* 2004; Pyšek *et al.* 2008). Natural experiments also tend to lack true replicates, which can make them limited and context dependent (e.g. different source pools, different environments, introductions at different times). Such problems can be avoided in manipulative experiments.

Provenance tests, largely known by ecologists as common garden experiments, provide the best of both worlds for studying invasions, as they are controlled experiments. However, if planted and left unchecked for long periods of time, they could initiate an unintended natural experiment of invasion (Zenni *et al.* 2014; Zenni and Hoban 2015; Zenni *et al.* 2016). For instance, provenance trials around the globe have shown the interaction existing between growth of loblolly pine (*Pinus taeda*) genotypes and environmental conditions (Falkenhagen 1978; Shimizu and Higa 1981; Schmidtling 1994; Harms *et al.* 2000; ZhiGang 2000). In China, there were significant variations among 15 different seed sources of loblolly pine and these variations were significantly correlated with minimum temperature in the seed origin (ZhiGang 2000). In South Africa, provenances of *P. taeda* from coastal southern Texas, southern Louisiana and Florida presented growth rates up to twofold greater than some other provenances (Falkenhagen 1978). In this case, strong correlations were found between growth in South Africa and latitude of the place of origin in North America. In Brazil, all provenance trials showed remarkable growth differences among provenances, likely having a climatic origin (Baldanzi *et al.* 1974; Baldanzi and Malinovski 1976; Shimizu and Higa 1981). As with loblolly pine, many other tree species have been planted around the world in forestry provenance trials and show similar trends of heritability for traits associated with greater invasive potential (e.g. Gapare *et al.* 2012a; Gapare *et al.* 2012b). Moreover, it is not uncommon that these experiments last for decades.

Researchers have been using provenance trials to understand species responses to climate change (i.e. Schmidtling 1994; O’Neill *et al.* 2008). Accordingly, such large scale, long-term experiments can be used to improve our understanding of non-native species and populations responses to new environments. Because provenance trials include a significant pool of seed sources and genetic lineages, researchers can use these experiments to detect intra-specific trait differences. In some cases, where the species escapes the experiment and starts invading surrounding areas, researchers can use available data from the experiment to test evolutionary processes related to naturalization and invasion (Zenni *et al.* 2014; Zenni and Hoban 2015; Zenni *et al.* 2016).

## Evolutionary mechanisms underlying tree invasions

Below, we explore evolutionary processes that have already been shown to occur during tree invasions and explain where and how they align with each barrier of the unified framework (Fig. 1). Our aim was twofold (i) to review current knowledge on how evolutionary processes affect tree invasions and (ii) suggest research strategies to further advance our understanding of how evolution affect tree invasions (e.g. Box 1).

### Pre-introduction evolutionary history

Frameworks for biological invasions depict the transportation of non-native individuals into a new range as a starting point for the processes leading up to establishment and subsequent invasion (Blackburn *et al.* 2011; Foxcroft *et al.* 2011; Gurevitch *et al.* 2011). However, the influence of evolutionary processes on invasion success may be manifest prior to transportation to a new region (Fig. 1; Thompson *et al.* 2012). Local adaptations and co-evolved symbiotic interactions developed in the native range can be key factors fostering or hindering invasive potential in new ecosystems (Lachmuth *et al.* 2010; Zenni *et al.* 2014). Variation in biotic and abiotic conditions across the landscape can promote differentiation across groups of individuals in their native ranges, potentially resulting in genetic differentiation and, ultimately, local adaptation (Mosca *et al.* 2012). Most life forms show intra-specific trait variation across their native populations. Coincidently, successful plant invaders often have wide native ranges (Hayes and Barry 2008). For instance, loblolly pine (*Pinus taeda*) shows considerable genetic variation in seed dormancy and climatic conditions needed for successful germination in its native range (Schultz 1997). For this species, seed size, seed weight and seed coat thickness vary by region and affect seedling growth (Schultz 1997). Similarly, two distinct provenances of Chinese tallow tree (*Triadica sebifera*) differ in cold hardiness, germination rate and overwinter seedling survival (Park *et al.* 2012) and appear to be genetically distinct (DeWalt *et al.* 2011). In some *Eucalyptus* species, phenotypic plasticity in response to drought depends on the origin of populations, with those from harsher climates exhibiting greater plasticity (Drake *et al.* 2015).

Trees commonly experience high seed and seedling mortality owing to maternal effects, stochasticity in seed dispersal, predation, damping-off pathogens or competition for resources. The ability of individual trees to overcome early mortality depends in large part on their inherited traits. Consequently, individual mother plants have differential contributions to future generations and the distribution of female reproductive success tends to be very skewed (Moran and Clark 2011). For example, in central Spain, 10 % of maritime pine (*Pinus pinaster*) trees mothered 50 % of offspring (González-Martínez *et al.* 2006), whereas for red oaks (*Quercus* spp.) in the eastern USA, <40 % of potential trees were estimated to have mothered at least one seedling (Moran and Clark 2011). In both these cases, bigger trees were more successful. Only a few genotypes survive past the seed stage in trees because the seed-seedling stages suffer a strong selection force. Consequently, the genetic characteristics of propagules sampled for transport (intentional or accidental) can strongly influence the invasive potential of a new population in the introduced range as detailed in the section below.

Pre-adaptations, such as those discussed above, are normally highly context (environment) specific (e.g. Zenni *et al.* 2014). Some genotypes are adapted to faster growth and earlier reproduction, making them more likely to naturalize if introduced to a new range (Matesanz and Sultan 2013). For example, the Neotropical tree *Miconia calvescens* has evolved high levels of phenotypic plasticity in response to light in its native range (Baruch *et al.* 2000). Under normal low-light and dense forest understory conditions, saplings develop slowly but respond rapidly to the availability of light when canopies open up, growing and developing rapidly into adult trees. This species harbours extremely low levels of genetic diversity throughout its invasive ranges (Le Roux *et al.* 2008; Hardesty *et al.* 2012); pre-adapted phenotypic plasticity to light availability likely plays a key role in this tree’s invasion success under less-saturated forest conditions in numerous Pacific Islands (Le Roux *et al.* 2008).

The long lifespan of many tree species make them ideal models to better understand the differences between native and introduced populations of particular species. In many instances, the original trees that initiated invasions are still living and their native provenances are known. This provides ideal opportunities to infer the contribution of evolutionary processes to invasion success under complex demographic scenarios (e.g. Zenni *et al.* 2014; Zenni and Hoban 2015). Comparative studies that consider ecological and evolutionary trajectories of populations descending from the same mother-plants located in both native and introduced ranges (i.e. reciprocal common gardens – see Box 1), could provide important insights into the importance of pre-adaptation, genotype–environment interactions, local adaptation, genetic drift and interactions with co-evolved mutualists for biological invasions and range shifts.

### Sampling effect

Species can be transported to a new range in several ways, accidently or intentionally. The means of, and reasons for, introductions will result in different propagule sampling for transport (Fig. 1). Sampling effects affect propagule pressure, including propagule diversity and therefore genetic diversity (sensu Zenni and Simberloff 2013). However, propagule pressure only considers the number and size of introduction events (Simberloff 2009) and does not take into consideration the genetic component of introductions although some have argued that genetic diversity is implicit in the concept (see Thompson *et al.* 2016). Propagule pressure is also influenced by life-history traits of different taxa (Colautti *et al.* 2006). The interplay between native genetic diversity and a species' introduction history ultimately determines the genetic diversity introduced (Le Roux *et al.* 2011). For instance, while ornamental trees tend to be transported in lower numbers and introduced in low densities, forestry species tend to be transported in higher numbers and introduced in high densities (Křivánek *et al.* 2006; Essl *et al.* 2010; McGregor *et al.* 2012; Donaldson *et al.* 2014; Crous *et al.* 2016). Furthermore, trees introduced for erosion control (e.g. *Pinus contorta* and *Alnus viridus* in New Zealand, *Prosopis* spp. in east Africa) are often transported in high numbers and introduced into particularly vulnerable habitats (Ledgard 1976; Wilson *et al.* 2009). Both ornamental and forestry pathways intentionally involve trait selections that are often linked with significant genomic changes like polyploidization or hybridization (Wilson *et al.* 2009; Essl *et al.* 2010). But the types of traits selected for in each pathway may differ drastically. While ornamental plants may be selected for flower, fruit, and foliage traits (Kitajima *et al.* 2006), forestry plants are usually selected for more rapid growth, disease resistance, and environmental hardiness (Carson and Carson 1989; Fenning and Gershenzon 2002; Wingfield *et al.* 2015). Artificially selected traits for ornamentation and forestry are also often positively linked to invasion success (Essl *et al.* 2010; Zenni 2014). There are also substantial efforts made to introduce and improve mycorrhizas for many forestry species (e.g. Ledgard 1976), which may cause substantial increases in tree invasiveness following fungal introduction (Wood *et al.* 2015). Thus, intentional introductions tend to be carefully planned (Box 1) and the chance of introducing individuals adapted to particular ecosystems is therefore high, as is the probability of establishment and spread (Essl *et al.* 2011).

Contrary to intentional sampling and transport mechanisms, many introduction events are accidental (Santini *et al.* 2013). In accidental transports, only a few propagules tend to be released (Wilson *et al.* 2009) and invasions are more likely to fail than to succeed (Zenni and Nuñez 2013). However, if high-performance genotypes or adapted individuals are present in the pool of accidentally released individuals, the chances of successful establishment may increase (Matesanz and Sultan 2013; Corliss and Sultan 2016). Organisms with some eco-evolutionary experience of the prevailing ecosystem conditions in the novel environment should have an inherent advantage to establish (Saul *et al.* 2013; Saul and Jeschke 2015). Unintentional introductions often suffer from severe founder events, reduced genetic diversity, and therefore strong genetic drift. This can lead to rapid, albeit non-adaptive (neutral), trait differentiation between introduced and native range populations (Keller and Taylor 2008). Once introduced, further range expansions by bottlenecked populations may exacerbate the effects of drift, and for small populations, drift may result in invasion failure (Zenni and Nuñez 2013). Strong founder events could also cause inbreeding and induce evolutionary change through the purging of genetic load (Facon *et al.* 2006; Schrieber and Lachmuth 2016).

Despite the importance of sampling in shaping the transported pool of propagules, the evolutionary histories and dynamics occurring in native ranges are not considered in the unified framework for biological invasions (Fig. 1). Many invasive tree species were introduced for economic and environmental alleviation purposes in which some level of intentional sampling of adapted species or genotypes was used (Castro-Díez *et al.* 2011; McGregor *et al.* 2012; Rejmánek *et al.* 2013). Thus, for tree invasions, it is important to consider sampling effects during the transport of propagules from one region to another to understand causes and mechanisms of invasion.

### Founder effects

The loss of genetic variation resulting from the sub-sampling and introduction of a small number of individuals from a source population, known as founder effect, suggests small populations recently introduced are unlikely to succeed (Kanarek and Webb 2010). Such reductions in genetic diversity may be exacerbated once a species starts to spread in the new environment. Higher genetic diversity in pools of introduced organisms is expected to allow the expression of a wider range of trait values compared to introduced pools characterized by genetic bottlenecks (Fig. 1). More variable populations tend to tolerate a wider range of environments and disturbances and have more phenotypes upon which natural selection can act. They are also more likely to survive attack by pests and pathogens, either introduced or native, than obligatory clonal populations or those with a narrow genetic base (Levin 1975; Clay and Kover 1996). However, genetic diversity may not be crucial for the successful establishment and spread of non-native populations, with many plant invasions caused by single genotypes and clonal plants (e.g. Le Roux *et al.* 2007; Hardesty *et al.* 2012). If an initial pool of individuals successfully survives and reproduces (naturalizes; Fig. 1), the population tends to accumulate new genetic diversity (Kanarek and Webb 2010). Additional introductions of propagules from the native range may also occur, possibly from distinct populations, or multiple independent introductions can coalesce over time in the new range (Dlugosch and Parker 2008). Further, mutations normally accrue at low rates and tend to be silent or detrimental, thus having no effect or a largely negative effect, with the latter usually being under strong negative selection in small populations. The larger the population becomes, the greater the chances are that some of the genotypes will successfully produce viable offspring.

Founder effects, just like sampling effects, although key in forming the genetic make-up of introduced populations, are not considered in the unified framework (Fig. 1). Tree invasions may help biologists understand the relative importance of founder effects in invasions by comparative analyses of multi-generational tree populations that are invading or failing to invade, or are in different stages of spread.

### Genotype × environment interactions

Most species introductions or colonizations result in establishment failure (Zenni and Nuñez 2013). This is possibly owed to mismatches between adaptations of introduced individuals acquired in their native range and the selective forces present in their newly introduced environments. However, for biological invasions, where introduced populations are often non-randomly sampled, the initial selection of pre-adapted or high-performance genotypes for introduction may contribute to rapid post-introduction adaptations (Matesanz *et al.* 2012; Matesanz and Sultan 2013; Corliss and Sultan 2016). These adaptations can increase survival and fitness of individuals, potentially leading to increases in population size, dispersal, and invasiveness (Colautti and Barrett 2013; Colautti and Lau 2015).

For many tree invasions, positive genotype-by-environment (G×E) interactions mediated by humans are key factors underlying invasion success. For instance, many forestry species were first introduced in provenance trials (Box 1) where foresters actively sought genotypes adapted for specific climatic conditions (Falkenhagen 1978; ZhiGang 2000). Once suitable genotypes were identified, they were selected for large scale planting (Box 1). The same active search for positive G×E interactions exists for horticultural and forage plants (Cook *et al.* 2005; Ebeling *et al.* 2011). Such positive G×E interactions may help introduced trees survive, reproduce, and spread (Fig. 1). In the unified framework, genetic diversity helps populations overcome selection pressures and transition from naturalized to invasive by potentially increasing fitness of populations (Fig. 1).

### Admixture, hybridization and polyploidization

During establishment and spread some populations might accumulate new diversity by recombination of standing genetic diversity, especially through admixture (intra-specific breeding between historically isolated populations) or hybridization (interbreeding between different species; Fig. 1). Further, additional introductions of propagules from the native range can occur, possibly from distinct native populations, also adding new diversity.

Admixture is a common phenomenon during introduction, establishment and spread of introduced organisms (Fig. 1; Ellstrand and Schierenbeck 2000). However, we lack evidence that admixture increases invasiveness (Rius and Darling 2014) but see Lavergne and Molofsky (2007). For invasive *P. taeda* populations, frequent admixture did not result in increased invasiveness (Zenni *et al.* 2014; Zenni *et al.* 2016). It remains unclear if the importance of admixture in invasiveness can increase over time as new genetic combinations occur in the population and selection acts on these. Because many tree species form populations with overlapping generations, they represent ideal systems to assess the frequency of admixed plants across multiple generations, without having to follow populations over time. Many invasive trees have been introduced to multiple locations for provenance × environment trials and therefore, post-introduction admixture resulting in elevated levels of genetic diversity could be a common, but hitherto overlooked, phenomenon.

Polyploidy has been repeatedly linked with plant invasion success (te Beest *et al.* 2011). On the one hand, polyploids are more commonly represented as invasives. On the other hand, for species with varying ploidy levels, invasive populations are more commonly polyploid. The mode of polyploidization, i.e. auto- or allopolyploidy (both processes associated with many plant breeding programs), greatly impacts the rate and direction of genetic and epigenetic changes (te Beest *et al.* 2011). Significant epigenetic changes (see below) and subsequent phenotypic effects have been reported for allopolyploids (Paun *et al.* 2010). Therefore, hybridization followed by genome multiplication (i.e. allopolyploidization) rather than autonomous genome multiplication *per se* (i.e. autopolyploidization) may lead to substantial epigenetic variation in polyploid genomes.

For introduced trees whole-genome duplication, through polyploidization, might be a particularly important evolutionary mechanism (Gaskin 2016). Polyploidization is often employed in forestry species because of the immediate and sometimes large phenotypic effects such as increased growth rate, denser wood and resistance to pathogens. In addition to these traits that could directly promote invasiveness, polyploidization also confers immediate genetic advantages linked to genetic diversity and gene expression, physiological and environmental tolerance, and altered biotic interactions (te Beest *et al.* 2011). For example, tetraploid *Acacia mangium*, a member of a highly invasive genus, had lower seed set compared to diploid genotypes, but had higher levels of self-compatibility, which can facilitate naturalization and invasion (Griffin *et al.* 2012).

In the unified framework, admixture, hybridization and polyploidization can affect the establishment of introduced plants, allowing the expression of novel phenotypes with increased fitness compared to source populations (Fig. 1).

### Rapid evolution

Introduced populations can evolve rapidly in response to selection pressures in the new environment (Bossdorf *et al.* 2005; Dlugosch and Parker 2008; Whitney and Gabler 2008; Moran and Alexander 2014). Rapid evolution in growth and productivity traits in long-lived trees can occur <50 years after introduction (Zenni *et al.* 2014; Zenni and Hoban 2015). For example, following the introduction of *P. taeda* just over 40 years ago to southern Brazil, evolutionary changes in plant growth rate and leaf traits are correlated with greater rates of spread of invasive populations (Zenni *et al.* 2016). Similarly, invasive populations of *Acer negundo* exhibited genetic differentiation in traits related to growth, leaf phenology and ecophysiology, providing evidence that genetic effects may have influenced the spread of established populations (Lamarque *et al.* 2015). Seedlings of invasive populations of the Siberian elm (*Ulmus pumila*) produce more biomass and can more efficiently allocate belowground resources towards growth than native populations under common garden conditions. This suggests evolutionary shifts of early life cycle traits (germination and growth of seedlings/saplings) in non-native *U. pumila* populations (Hirsch *et al.* 2012; Hirsch *et al.* 2016). Despite these examples, there are very few case studies of rapid evolution for long-lived trees compared to the large number of successful tree invaders. For herbaceous plants, these patterns have been examined more frequently since their short life cycles more easily facilitate experimentation (e.g. Colautti and Barrett 2013; Turner *et al.* 2014; Colomer-Ventura *et al.* 2015).

In the unified framework (Blackburn *et al.* 2011), rapid evolution is a definite mechanism that can allow populations to transition from the establishment to spread stages (Fig. 1), specifically by decreasing the limitations of stochastic effects on invasiveness (Kanarek and Webb 2010). Invasive trees may make a less suitable group to study rapid evolution. Trees, however, often produce abundant seeds and suffer great selection pressures at the seedling stage. The major difficulty for studies of rapid evolution in trees is to follow the fate of at least several generations in order to detect genetic changes in populations (Box 1).

### Epigenetics

The modern evolutionary synthesis, based on the assumption that only heritable genetic variation and its origin by random mutation explain evolution by natural selection, is challenged by the rapidly expanding field of epigenetics (Feinberg and Irizarry 2010). Epigenetics involves molecular mechanisms that can cause variation in gene expression levels (and thus trait variation) without changes in the underlying DNA sequence (Richards 2006). In this regard, recent research has shown that epigenetic variation, e.g. DNA methylation profiles, can be heritable over multiple generations (Verhoeven *et al.* 2010).

Despite the potential impact of epigenetics on natural selection, the link between epigenetic variation and functional genomics remains weak. This is complicated by actual genetic diversity between individuals and/or populations, making inferences of epigenetic versus genetic contributions to phenotypic trait variation difficult. Moreover, environmentally induced epi-alleles may not be heritable and therefore would contribute only to pure phenotypic plasticity rather than to adaptive variation (Richards *et al.* 2010). Epigenetic variation may benefit non-native species, especially when the introduced standing genetic variation is low (Schrey *et al.* 2012). Epigenetically controlled traits are usually differentially expressed in response to environmental cues and, when heritable, could have significant adaptive value, even in the absence of the initial environmental stressor (Johnson and Tricker 2010). As previously mentioned, newly introduced populations often experience genetic bottlenecks and/or are exposed to novel environmental conditions. These species might consequently experience increased epigenetic variation as invasion progresses along the naturalization–invasion continuum.

Insights into the role of epigenetics in invasion biology, while rare and almost non-existent for trees, are now emerging. Wilschut *et al.* (2016) recently found heritable trait variation (flowering time) to be correlated with heritable epi-alleles across different populations of an apomictic clonal lineage of common dandelion (*Taraxacum officinale*). Similarly, the successful invasion of diverse habitats by genetically depauperate populations of Japanese knotweed (*Fallopia japonica*) appears to be correlated with epigenetic differentiation (Richards *et al.* 2012). Further, as discussed, hybridization sometimes precedes invasiveness (Ellstrand and Schierenbeck 2000) and the combination of diverged parental genomes in hybrids often requires major epigenetic modifications to re-establish compatibility between divergent parental genomes (Rieseberg 2001). For example, in invasive *Spartina anglica* populations, methylation changes are predominantly associated with inter-specific hybridization and rarely with intra-specific genome doubling (Salmon *et al.* 2005).

Even though limited information is available on the role of epigenetic variation during establishment and spread of non-native species, the possible role of such diversity in compensating for the low genetic variability typical of most introductions is conceivable. When heritable, epigenetic variation can serve as a form of ‘molecular memory’ to optimize environmental compatibility through phenotypic plasticity (Wilschut *et al.* 2016). For example, in poplar trees (*Populus* spp.) genetically identical clones obtained from different geographic plantings showed differences in gene expression when exposed to drought stress under common garden conditions (Raj *et al.* 2011). These differences reflect divergences in DNA methylation patterns between geographic provenances, with those provenances with longer residence times (time since planting) having the most distinct location-specific patterns in gene expression response (Raj *et al.* 2011).

Introduced trees represent particularly interesting systems to study the role of epigenetics in invasion success. As mentioned, trees are usually long-lived with many species having complex life cycles; they must therefore cope with variable environmental conditions over long and individual lifespans (Rohde and Junttila 2008), which can limit natural selection under fluctuating environmental conditions over short timescales (sensu Davis *et al.* 2005). High levels of phenotypic plasticity are therefore seen as a vital strategy to cope with these limitations, especially when standing genetic diversity is low, as is the case for many introduced species.

### Second-genomes

Like most higher organisms, almost all tree species rely on mutualistic interactions for nutrient acquisition. Consequently, they often require either the formation of novel associations with native mutualists or co-introduction with invading partners (Nuñez and Dickie 2014; Burgess *et al.* 2016). Concurrently, both the initial absence and subsequent accumulation of symbiotic microbes (Diez *et al.* 2010), comprising myriad fungi, oomycetes, bacteria and viruses, can be a key determinant of invasiveness (Crous *et al.* 2016). The phenotype of an organism is not determined solely by the interaction of the genotype and the environment and its epigenome but also by the combined genomes of closely physiologically associated symbionts. These have been termed ‘second-genomes’ (Grice and Segre 2012), recognizing that physiological traits controlled by symbiotic genomes are often overlooked as being neither classically ‘genomic’ nor entirely encompassed within environmental acclimation. Second-genomes comprised of closely associated symbionts can be a critical and semi-heritable (transmitted by the parents but also environmentally determined) determinant of plant adaptive traits (e.g. Rout and Southworth 2013).

We consider second-genome interactions as distinct from most biological interactions (e.g. competition, generalist pollination, seed dispersal and herbivory) because the duration of interaction between individual host plant and symbiont generally spans a substantial portion of the life cycle of at least one interacting partner. For example, infection of legume root hairs and the formation or endosymbiotic root nodules by rhizobia would be an instance where a second-genome associated with legume plants. Further, the symbiotic nature of second-genome interactions implies that species phenotype can be strongly determined by second-genomic controls, including phenology (Courty *et al.* 2007) and foliar traits (Moeller *et al.* 2016).

When plant species are introduced to a new range, their second-genome may be accidentally or intentionally co-introduced on plant tissue or in soils, leading to co-invasion (Dickie *et al.* 2010; Diez *et al.* 2010; Nuñez and Dickie 2014). Alternatively, introduced plants can form ‘novel associations’ with native symbionts or co-xenic novel associations (Nuñez and Dickie 2014) can be formed between symbionts from different regions (Klock *et al.* 2015; Le Roux *et al.* 2016). The level of interaction specificity, coupled with chances of co-introduction, may therefore have serious fitness consequences for introduced plants. Pines, for example, largely rely on co-introduced mycorrhizal fungi in New Zealand, South America and Hawaii (Hynson *et al.* 2013; Hayward *et al.* 2015) for establishment and growth; however, novel associations are common in pine plantations in Iran (Bahram *et al.* 2013). *Eucalyptus* in Europe appear to be largely associating with co-introduced symbionts (Diez *et al.* 2010), but where introduced as seeds without symbionts in the Seychelles they appear to form novel associations (Bahram *et al.* 2013); in New Zealand co-xenic associations (both interacting partners non-native) between *Eucalyptus* and European *Amanita muscaria* have also been observed (Nuñez and Dickie 2014). Similarly, the symbioses between legumes and rhizobia provide additional examples of co-introductions. For example, invasive Australian wattles (genus *Acacia sensu stricto*) appear to have been co-introduced with their Australian rhizobia to South Africa (*Acacia pycnantha*, *Aacacia mearnsii, Acacia saligna* and *Acacia longifolia*) (Ndlovu *et al.* 2013; Le Roux *et al.* 2016), Europe (*A. longifolia*, *A. saligna*) (Rodríguez-Echeverría *et al.* 2009, 2012), and New Zealand (*A. longifolia*) (Weir *et al.* 2004). In addition, there is evidence that acacias that have become invasive in multiple parts of the globe are more promiscuous hosts (i.e. capable of associating with a vast diversity of rhizobial mutualists) (Klock *et al.* 2015), potentially facilitating their invasion in novel ranges. Finally, the rates of association and the benefits from mycorrhizal associations may differ between native and invasive trees, providing a competitive advantage to invasive trees. For instance, *T. sebifera* exhibits higher degrees of arbuscular mycorrhizal colonization compared to native species, which may partly explain the successful invasion of the species into coastal plant communities of the southeastern USA (Paudel *et al.* 2014).

Pathogens can also co-invade, potentially hampering plant invasion success. For example, *Diplodia sapinea*, an important Botryosphaeriaceae conifer pathogen, has been introduced into every country where *Pinus* spp. are propagated as exotics (Burgess and Wingfield 2002). This pathogen is also horizontally transferred to trees after germination (Bihon *et al.* 2011). *Diplodia sapinea* is almost exclusively limited to conifers and mainly *Pinus* species; other species in the Botryosphaeriaceae show preference for angiosperm hosts (De Wet *et al.* 2008). Fungi in the Botryosphaeriaceae are particularly interesting because they are common endophytes in woody plants studied and they include important latent pathogens; further work is needed to determine how widely they are co-introduced and co-invade. Co-invading pathogens and native pathogens on invasive trees can transfer to native plants (spillover and spillback), also contributing to tree invasions (Blackburn and Ewen 2016).

A lack of compatible mutualists may also be a factor in invasion failures (Zenni and Nuñez 2013). For the genus *Casuarina*, invasion is limited by a lack of mutualistic and nitrogen-fixing *Frankia* bacteria (Zimpfer *et al.* 1999). Similarly, for legume–rhizobium associations, the importance of co-introductions for establishment success is exemplified by *Mimosa pudica* introductions to India. Here, invasive populations of *M. pudica* only nodulate with co-introduced South American rhizobia and appear unable to utilize rhizobia associated with sympatric, but native, Indian *Mimosa* species (Gehlot *et al.* 2013). Besides horizontal transfer, some plant symbionts are capable of transmission directly from mother plant to seed, and hence spread along with the plant. However, most do not and must therefore spread independently from the plant host. For mutualisms, this creates a chicken-and-egg paradox: which comes first? The plant or its mutualists? In the case of pine invasions, it appears that as little as a single individual of the fungus *Suillus luteus*, which is adapted for animal dispersal and a critical pine mutualist, may facilitate pine establishment (Hayward *et al.* 2015; Wood *et al.* 2015). Neither the pine, nor the fungus, may be able to invade without the other, and it is unclear which one allows the spread of the other.

Very little is known about second-genomes, particularly in the context of invasion. Most work on invasive tree second-genomes has focused on identification of symbionts, with less understanding of how these associated microorganisms might influence the tree physiology or invasiveness (but see Dickie *et al.* 2016; Klock *et al*. 2016). Increased introduction effort of other members in a plant genus or family may be more important in overcoming the constraints associated with second-genomes than introduction effort of a particular species.

## Conclusions

Organisms transitioning from casual to invasive are called ‘invasive non-native species’; however, the dynamics underlying these processes are likely led by a few individuals and occur at the population level (Corliss and Sultan 2016; Zenni *et al.* 2016). The concept of pre-adaptive experience in novel environments is particularly helpful to articulate the ecological and evolutionary mechanisms behind the inherent establishment ability of a plant, or its outright failure to spread. For long-lived species such as trees, in particular, higher eco-evolutionary experience, coupled with the ability to adapt *in situ*, can be a powerful framework to help explain their long-term persistence in landscapes. Indeed, integrating genetics with population dynamics is critical to better understanding the probability of either niche conservatism or niche evolution of an invader species in time (Holt 2009; Lavergne *et al.* 2010). This is especially important since species niches are considered to be under constant natural selection pressures, and thus a lack of adaptive traits in the novel environment might not be as generalizable as previously thought (Lavergne *et al.* 2010; Suda *et al.* 2015).

Demographic, genetic and environmental factors, and their stochasticity, interact to determine a population’s invasion success. Possessing genes that increase fitness at a given site can be critical for the survival, growth and reproduction of newly introduced populations. Recently introduced individuals, as well as individuals at the leading edge of an invasion front during the spread stage, are also likely to have more depauperate mutualist communities than the trailing edge of the population as the ability to associate with local microorganisms is limited. Thus, evolutionary factors, including the second-genomes, are key mechanism of invasions. The same may apply to standing genetic variation, with erosion of genetic diversity as spread progresses across landscapes. Many of the population-level processes undergone by invasive populations, as described in our additions to the unified framework (Fig. 1), have strong evolutionary roots.

The unified framework for biological invasions (Blackburn *et al.* 2011) provides a useful general description of the invasion process. However, it does not suggest mechanisms to breach the stages and barriers associated with invasion or explicitly consider a species’ or population’s eco-evolutionary history prior to introduction. Here, we have explicitly addressed these points in order to expand and unify how these mechanisms may affect plant invasions, with a focus on trees. Other non-evolutionary factors also greatly affect biological invasions, which have been discussed at great length in the scientific literature (i.e. Van Kleunen *et al.* 2010; Lamarque *et al.* 2011; Speek *et al.* 2011). Similarly, other eco-evolutionary frameworks have been proposed to improve our understanding of biological invasions (i.e. Facon *et al.* 2006; Saul *et al.* 2013; Saul and Jeschke 2015).

Our goal was not to propose a new framework, or even an expanded version of the existing unified framework. Rather, we have sought to populate a general framework with potential evolutionary mechanisms affecting the invasion stages and barriers (Fig. 1). By highlighting these mechanisms, researchers should become better equipped to understand and manage the observed patterns of biological invasions.

## Sources of Funding

This paper had its origin at a workshop on ‘Evolutionary dynamics of tree invasions’’ hosted by the Department of Science and Technology - National Research Foundation Centre of Excellence for Invasion Biology (CIB) in Stellenbosch, South Africa, in November 2015. Funding for the workshop was provided by the CIB, Stellenbosch University (through the office of the Vice Rector: Research, Innovation and Postgraduate Studies), and the South African National Research Foundation (DVGR grant no. 98182). RDZ was funded by Conselho Nacional de Desenvolvimento Científico e Tecnológico (grant number 313926/2014-0). IAD was funded by CORE funding to the Bio-Protection Research Centre. TGZ acknowledges funding from Coordenação de Aperfeiçoamento de Pessoal de N**í**vel Superior (CAPES). CC thanks the members of the Tree Protection Co-operative Programme (TPCP) and the DST-NRF Centre of Excellence in Tree Health Biotechnology at FABI, University of Pretoria, for funding. JLR acknowledges funding from South Africa's National Research Foundation (grant no. 93591). 

## Contributions by the Authors

RDZ, ID and JLR conceived the original idea and led the writing with substantial contributions from MW, HH, LM and TB. CC, TGZ, MK, ES, AE, AR and LM contributed text and ideas to specific parts of the manuscript. All authors contributed to discussions and subsequent writing of the manuscript.

## Conflicts of Interest Statement

None declared.
